# Inverted Y‐shaped technique for complex superior mesenteric / portal vein reconstruction in pancreatoduodenectomy for locally advanced pancreatic head ductal adenocarcinoma

**DOI:** 10.1002/ags3.12666

**Published:** 2023-02-20

**Authors:** Benson Kaluba, Naohisa Kuriyama, Takahiro Ito, Akihiro Tanemura, Shugo Mizuno

**Affiliations:** ^1^ Department of Hepatobiliary Pancreatic and Transplant Surgery Mie University Graduate School of Medicine Tsu Japan

**Keywords:** pancreatoduodenectomy, superior mesenteric/portal vein axis, vascular resection and reconstruction

## Abstract

Most pancreatoduodenectomy (PD) procedures for locally advanced pancreatic head adenocarcinoma (PDAC) require superior mesenteric/portal vein (SMV/PV) axis resection and reconstruction. Here we describe the inverted Y‐shaped as a new technique for complex SMV/PV reconstruction and aimed at evaluating its safety and effectiveness. Among 287 patients who underwent PD for locally advanced PDAC from April, 2007 to December, 2020 at our hospital, 11 patients (3.8%) who underwent PV/SMV reconstruction with this technique were enrolled. Briefly, two distal veins were slit‐wedged, sutured, resulting in one orifice, then reconstruction was completed with (*n* = 6) or without (*n* = 5) interposed autologous right external iliac vein (REIV) grafts, respectively. Operation time and blood loss were 649 (502–822) min and 1782 (475–6680) mL, respectively. The median length of resected SMV/PV was 40 (20–70) mm, 50 (50–70) mm for REIV grafts, and the splenic vein was resected in eight patients. No patient developed pancreatic fistula; mild leg edema was observed in the six graft patients and the median hospital stay was 36.0 d. PV patency rate at 2 mo after PD was 91% (10/11) and no 90‐d mortality was recorded. R0 resection rate was 91% (10/11). It is feasible to safely reconstruct the SMV/PV using the inverted Y‐shaped technique in appropriately selected PDAC patients.

## Graphical abstract

We describe the inverted Y‐shaped as a new technique for complex superior mesenteric/portal vein reconstruction. This reconstruction technique is safe and effective in appropriately selected pancreatic head adenocarcinoma patients.

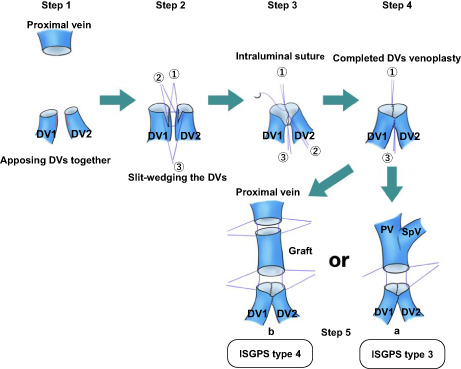

## 1 INTRODUCTION

Pancreatic ductal adenocarcinoma (PDAC) is an aggressive solid tumor with a mortality rate that nearly equals its incidence due to early lymphovascular and perineural invasion.^1^ Consequently, most patients at initial diagnosis have advanced unresectable‐metastasized (UR‐M) tumors, which involve major vessels such as the superior mesenteric / portal vein (SMV/PV) and celiac axis / superior mesenteric artery (CA/SMA).^2^

Current treatment uses a multidisciplinary approach that combines neoadjuvant/adjuvant therapy and pancreatectomy. Neoadjuvant therapy is associated with improved chances of negative (R0) resection, the only cure that leads to improved patients' overall survival.^3^ When PDAC of the pancreatic head infiltrates the SMV/PV axis, pancreatoduodenectomy (PD) combined with SMV/PV resection and reconstruction is performed, a procedure first reported by Fortner et al.^4^ Based on the International Study Group of Pancreatic Surgery (ISGPS) classification,^5^ there are four vascular reconstruction types (venorrhaphy, venoplasty with a patch, primary veno‐venous anastomosis, and anastomosis with an interposed venous graft).

This study aimed at evaluating the safety and efficacy of reconstructing the SMV/PV axis following PD for locally advanced PDAC with an inverted Y‐shaped (double‐single orifice) technique. Surgical steps for this technique are described.

## 2 MATERIALS AND METHODS

### 2.1 Patients

Of 287 patients who underwent PD for locally advanced PDAC, 11 (3.8%) who had SMV/PV axis resection and reconstruction with the inverted Y‐shaped technique between April 2007 and December 2020 at our hospital were enrolled. Table 1 shows their characteristics.

**TABLE 1:** Patient characteristics

Variable	Patients (*n* = 11)
Preoperative characteristics	
Male/Female	5/6
Age (y)	70.0 (50–85)
Body mass index (kg/m^2^)	20.4 (15.1–25.5)
ASA classification (1/2/3/4)	2/7/2/0
Initial resectability classification (R/BR‐PV/BR‐A/UR‐LA)	2/4/2/3
Neoadjuvant therapy	10 (90.9)
Intraoperative findings	
Operation time (min)	649 (502–822)
Blood loss (mL)	1782 (475–6680)
Pancreatic parenchyma texture (soft/hard)	2/9
MPD diameter (≤3 mm/>3 mm)	2/9
Resected length of SMV/PV (mm)	40 (20–70)
PV diameter (mm)	10 (7–12)
DV1 diameter (mm)	6.3 (4.3–7.8)
DV2 diameter (mm)	4.8 (3.6–7.1)
Interposed venous graft using REIV	6 (54.5)
Venous graft length (mm)	50 (50–70)
Venous graft diameter (mm)	10.9 (10.4–14.0)
SMV/PV reconstruction ISGPS type (3/4)	5/6
Combined splenic vein resection	8 (72.7)
Postoperative outcomes	
Clavien–Dindo (max) (0/1/2/3b/4b)	4/5/1/1
Postoperative pancreatic fistula	0
Abdominal abscess	3 (27.2)
Biliary leakage	1 (9.1)
Postoperative pancreatic hemorrhage	1 (9.1)
Delayed gastric empty	2 (18.2)
Leg edema	6/11
Hospital length of stay (d)	36.0 (21–105)
90‐d mortality	0
Pathological findings (UICC 8th)	
Tumor size (mm)	29 (14–63)
Tumor differentiation (G1/G2/G3)	4/3/4
T factor (T3/T4)	10/1
N factor (N0/N1/N2)	9/2/0
M factor (M0/M1)	11/0
R0/R1 resection	10 (90.9)/1 (9.1)
Assessing SMV/PV anastomosis patency	
Short‐term patency (<30 d)	10 (90.9)
Long‐term patency (>30 d)	10 (90.9)

*Note*: Data are expressed as number (percentage), median (range).

Abbreviations: ASA, American Society of Anesthesiologists; BR‐A, borderline resectable artery; BR‐PV, borderline resectable portal vein; DV, distal vein; ISGPS, International Study Group of Pancreatic Surgery; MPD, main pancreatic duct; R, resectable; SMV/PV, superior mesenteric vein/portal vein; UICC, Unio Internationalis Contra Cancrum; UR‐LA, unresectable‐locally advanced.

### 2.2 Surgical procedure of SMV/PV axis resection and reconstruction with the inverted Y‐shaped technique

**FIGURE 1:**
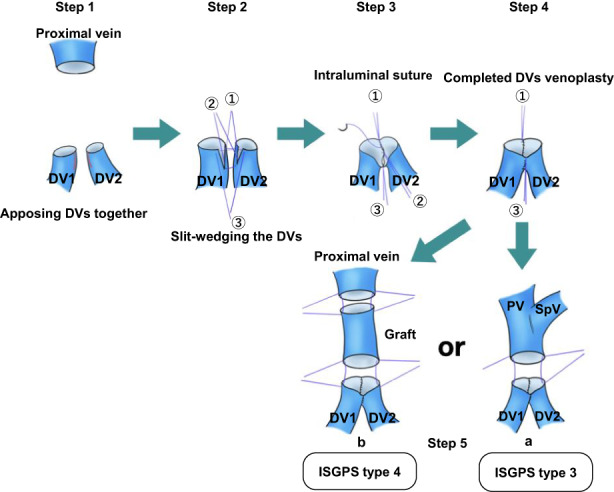
Surgical steps of the inverted Y‐shaped reconstruction technique. DV1 and DV2 were each held by a vascular clamp and apposed side‐to‐side (Step1), then they were slit‐wedged on the sides facing each other (Step 2). 6‐0 vascular sutures were placed as the posterior ①, central ②, and anterior ③ stay sutures. The posterior one was secured in a surgical knot with a needle thread which was then used to perform an intraluminal suture from the posterior to the central side. On reaching the central side, the needle thread and posterior stay suture were gently pulled in opposite directions, allowing for growth factor of the new orifice. The same procedure was repeated from the central to the anterior side (Step 3). Finally, the needle thread was secured in a knot with the anterior stay suture and venoplasty of DV1 and DV2 was completed (Step 4). For end‐to‐end anastomosis, intraluminal suturing was used for the posterior wall and the over‐and‐over suturing for the anterior wall. In the five patients without grafts (ISGPS type 3), the new venoplasty orifice was directly anastomosed to the proximal vein (Step 5a). For the graft patients (ISGPS type 4), a distal anastomosis was first performed between the caudal orifice of the interposed REIV and the new venoplasty orifice of anastomosed DV1 and DV2, after which the ventral orifice of REIV was then anastomosed to the proximal vein (Step 5b). DV1, distal vein 1; DV2, distal vein 2; ISGP, International Study Group of Pancreatic Surgery; PV, portal vein; SpV, splenic vein.

After an upper‐midline incision, our standard anterior approach to the SMA was used as described by Mizuno et al.^6^ After measuring the involved length of the SMV/PV axis, the pancreatic head together with the tumor‐involved SMV/PV segment was resected (Video S1). Figure 1 highlights the steps of the vascular reconstruction. In six patients, sufficient lengths of autologous right external iliac vein (REIV) grafts were harvested for inter‐positioning (Video S1 and Table 2). Two distal branches of SMV (DV1 and DV2) were each held by a vascular clamp apposed side‐to‐side (Step 1), then, depending on the diameter of the veins, slit‐wedges of no longer than 3 mm were made on their medial (DV1) and lateral (DV2) opposite sides to increase the size of the anastomosis diameter (Step 2). To avoid tearing the thin walls of distal veins and minimize forceps‐handling of the veins, three stay sutures were gently placed (posterior, central, and anterior) of the apposed orifices for traction, then an intraluminal suture technique using 6‐0 vascular sutures from the posterior to the central side was employed. Upon reaching the central side, the needle‐thread and posterior stay suture were gently pulled in opposite directions, allowing for the growth factor of the new orifice. The same procedure was repeated from the central to the anterior side (Step 3). Finally, the needle thread was secured in a knot with the anterior stay suture and the venoplasty of DV1 and DV2 was completed (Step 4). Depending on the surgeon's preference, the venoplasty can be performed from the anterior to posterior side, as shown in Video S2. For end‐to‐end anastomosis, intraluminal suturing was used for the posterior wall and the over‐and‐over suturing for the anterior wall. In the five patients without grafts (ISGPS type 3), the new venoplasty orifice were directly anastomosed to the proximal vein (Step 5a). For the graft patients (ISGPS type 4), as shown in Video S3, a distal anastomosis was first performed between the caudal orifice of the interposed REIV and the new venoplasty orifice, after which the ventral orifice of REIV was anastomosed to the proximal vein (Step 5b). Following vascular reconstruction, the digestive tract and biliary tree were reconstructed using a modified Child method by performing end‐to‐side pancreatojejunostomy, end‐to‐side hepaticojejunostomy, and end‐to‐side or side‐to‐side gastrojejunostomy. In the postoperative period, anticoagulant therapy was not routinely used.

**TABLE 2:** Demographics and clinical data of patients with inverted Y‐shaped superior mesenteric / portal vein reconstruction technique

	Sex	Age	NAT	Initial resectability	OP time (min)	Blood loss (mL)	Diameter P‐V (mm) A	Resected L (mm) B	Diameter DV1 (mm) C	Diameter DV2 (mm) D	Graft	Graft L (mm) E	Graft diameter (mm) F	Complication	CDC	R0 resection	Hospital LOS (d)	<30 d patency	>30 d patency
1	F	68	Y	UR‐LA	746	6680	9 (PV)	70	7.8 (SMV)	6.2 (J2)	Y	50	14.0	Cholangitis	2	Y	105	Y	N
2	F	62	Y	BR‐PV	561	970	7 (SMV)	20	5.2 (SMV)	4.3 (J2)	N			DGE	2	Y	42	Y	Y
3	M	69	Y	BR‐PV	780	1900	12 (PV)	40	7.2 (SMV)	5.8 (J2)	N			N	0	Y	31	Y	Y
4	M	77	Y	BR‐A	529	2613	10 (PV)	40	6.3 (SMV)	4.5 (J2)	N			N	0	Y	21	Y	Y
5	F	50	Y	UR‐LA	694	1150	8 (PV)	50	5.4 (SMV)	4.5 (J2)	Y	50	10.6	IAA, BL	2	N (R2: DPM)	36	Y	Y
6	F	77	Y	UR‐LA	646	1250	12 (PV)	35	4.3 (SMV)	3.6 (J2)	Y	50	12.9	N	0	Y	38	Y	Y
7	M	77	Y	BR‐PV	822	1782	10 (SMV)	50	7.5 (SMV)	4.8 (J1,2)	N			N	0	Y	25	Y	Y
8	F	75	N	BR‐A	502	475	10 (SMV)	50	5.2 (SMV)	4.9 ((J1,2)	N			IAA, DGE	2	Y	21	Y	Y
9	F	85	Y	R	606	681	10 (PV)	40	6.2 (SMV)	6.9 (J1,2)	Y	50	10.4	N	0	Y	39	Y	Y
10	M	58	Y	BR‐PV	521	1814	12 (PV)	40	6.6 (SMV)	4.2 (J1,2)	Y	50	11.0	IAA, PPH, PE	4b	Y	48	Y	Y
11	M	71	Y	R	813	2864	11 (PV)	40	7.8 (SMV)	7.1 (J1)	Y	50	10.8	RC	3b	Y	34	N	Y

Abbreviations: BR‐A, borderline resectable artery; BR‐PV, borderline resectable portal vein; CDC, Clavien–Dindo classification; DGE, delayed gastric emptying; DPM, dissected pancreatic margin; DV, distal vein; IAA, intra‐abdominal abscess; J, jejunal vein; L, length; LOS, length of stay; N, No; NAT, neoadjuvant therapy; OP, operation; PE, pleural effusion; PPH, postpancreatectomy hemorrhage; PV, portal vein; P‐V, proximal vein; R, resectable; RC, respiratory complication; SMV, superior mesenteric vein; UR‐LA, unresectable‐locally advanced; Y, Yes.

### 2.3 Assessing SMV/PV anastomosis patency

Portal vein patency was assessed on postoperative dynamic computed tomography (CT)‐scan imaging done 6 d after surgery and in the follow‐up period.

## 3 RESULTS

### 3.1 Patients

As summarized in Tables 1 and 2, this study had six female and five male patients with a median age of 70.0 (50–85) y and BMI of 20.4 (15.1–25.5) kg/m^2^. Neoadjuvant therapy was administered to 10 (91.0%) patients. Initial resectability classifications were R (*n* = 2), BR‐PV (*n* = 4), BR‐A (*n* = 2), and UR‐LA (*n* = 3), respectively.

### 3.2 Surgical outcomes

Median operation time and blood loss were 649 (502–822) min and 1782 (475–6680) mL. Median resected length of SMV/PV was 40 (20–70) mm, splenic vein was resected in eight patients, and proximal veins were PV (*n* = 8) and SMV (*n* = 3) with a median diameter of 10.0 (7–12) mm. Median diameters of distal veins were 6.3 (4.3–7.8; DV1) mm and 4.8 (3.6–7.1; DV2) mm, while grafts had a median length of 50 (50–70) mm.

Cases 10 and 11 had clinically significant Clavien–Dindo (CD)^7^ postoperative complications of III or more. The former developed intra‐abdominal abscess, postpancreatectomy hemorrhage (PPH), and pulmonary effusion, while the latter had respiratory complications. Two patients (18.2%) developed delayed gastric emptying (DGE), while one had biliary leakage. However, mild leg edema in the six graft patients was observed, which resolved without any intervention, but no patient developed postoperative pancreatic fistula (POPF); the median hospital stay was 36.0 (21–105) d and no 90‐d mortality was recorded.

Tumor classification based on the 8th edition of AJCC/UICC TNM staging^8^ were T3 (*n* = 10), T4 (*n* = 1), and N0 (*n* = 9), N1 (*n* = 2). Lastly, R0 resection was achieved in 10 (91%) patients.

### 3.3 Assessing SMV/PV anastomosis patency

The SMV/PV anastomosis in all five nongraft patients remained patent even on dynamic CT scan done 2 mo after PD. Case 11 developed immediate PV thrombosis and a percutaneous PV stent was placed on postoperative d 1, which remained patent on follow‐up CT scan done 23 d later, as shown in Figure 3A–D. Anticoagulant treatment with low‐molecular weight heparin was commenced from postoperative d 1 to 6 before converting to oral anticoagulant medication for 6 mo. Case 1 developed SMV/PV anastomosis stenosis with significant collateral vein formation postoperative d 53, upon which oral anticoagulant treatment was started for 6 mo, as shown in Figure 3E–G.

## 4 DISCUSSION

Pancreatic head cancer easily invades the PV/SMV axis due to its anatomical relationship; therefore, resection and reconstruction of PV/SMV is an essential technique to achieve R0 resection in some PD cases.^3^ Here we propose the use of the inverted Y‐shaped technique to reconstruct the SMV/PV axis in PD procedures where long segments of SMV/PV are involved, leading to occlusion of distal SMV branches (jejunal and iliac veins). The technique allows for SMV/PV reconstruction involving proximal veins (with wider diameters) and two distal branches of SMV (with narrower diameters). Moreover, RIEV grafts should be considered for use in SMV/PV resection cases of ~30 mm or more.

**FIGURE 2:**
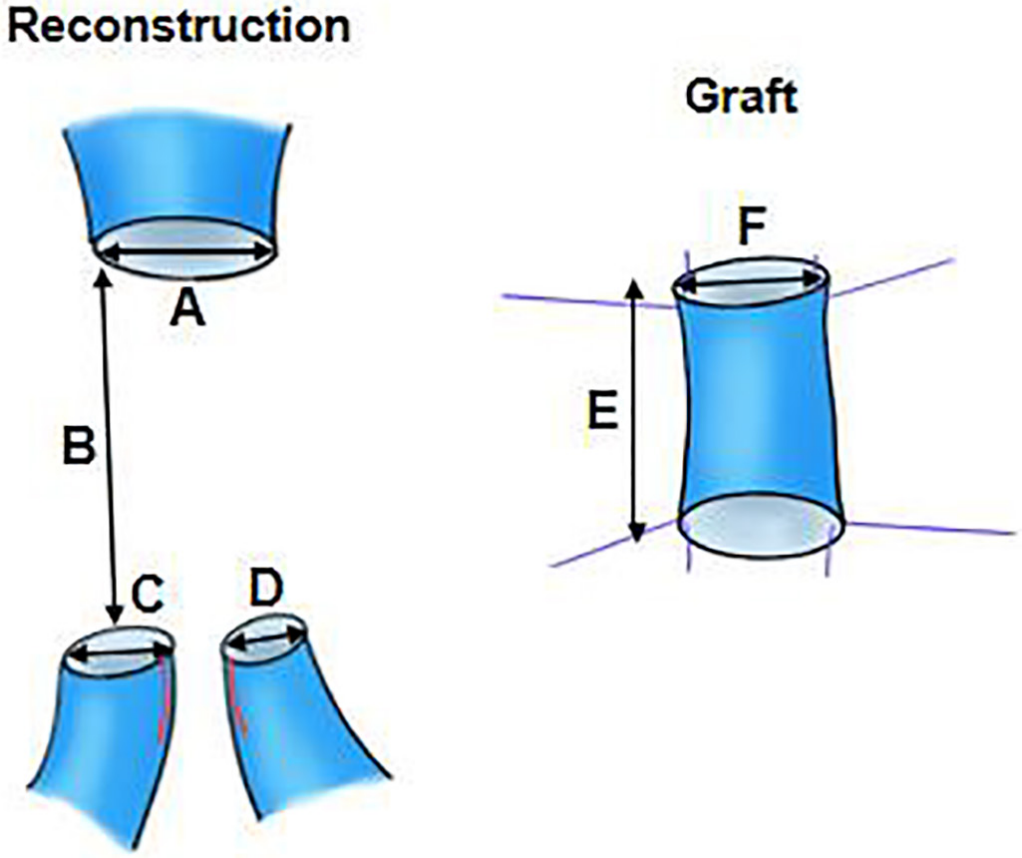
Venous lengths and diameters. (A) Diameter of proximal vein (mm), (B) resected length of superior mesenteric/ portal vein (mm). (C) Diameter of 1st distal vein (mm), (D) diameter of 2nd distal vein (mm). (E) graft length (mm), (F) diameter of graft (mm)

These PD cases are quite rare, but Oba et al^9^ reported a technique similar to ours (Figure 1). In their technique, the distal veins were not wedged and the new orifice still had partitions by the side‐to‐side anastomosis lines. We, however, slit‐wedged and then sutured the two distal veins to increase the diameter of the new orifice, which had no divisions, and solved the diameter size‐mismatch between the proximal veins and distal branches of SMV (DV1 and DV2) as shown in Table 1 and Figure 2.

Pancreatoduodenectomy is a complex procedure and intraoperative blood loss is frequently encountered, even by expert surgeons. Six (54.5%) of our patients had >1500 mL blood loss, but only Case 1 had massive bleeding, and the operating time in this one was more than in the other five patients. Even though most post‐PD complications are managed conservatively, they impact patient recovery, length of hospital stay, and medical costs.^10^ The three most common include POPF, DGE, and PPH with incidence rates of 22%–26%, 14%–30%, and 3%–10% respectively.^11^ In this study, POPF was not encountered; two (18.2%) had DGE while one (9.1%) patient developed PPH, yet only two (18.2%) had complications of CD‐III or more.^7^

The splenic vein was resected in eight patients without further reconstruction, but concomitant splenic artery (SA) ligation was done in one patient. Mizuno et al^12^ linked SpV division to variceal formation and thrombocytopenia leading to bleeding. Therefore, some recommend either SpV reconstruction^13^ or SA ligation^14^ to avoid left‐sided portal hypertension (LPH). However, Tanaka et al^15^ reported no significant benefits with reconstructing the SpV, while, Gyoten et al^16^ concluded that SA ligation did not prevent LPH, but just delayed its occurrence.

After SMV/PV resection, direct end‐to‐end anastomosis is safe for within a 20 mm length, while interposed grafts are usually used for >30 mm lengths.^17^ Terasaki et al^17^ performed 122 SMV/PV resections and compared outcomes of patients who underwent end‐to‐end (97/122) and RIEV graft interpositioning (25/122). Short‐ and long‐term outcomes were comparable between the two groups, but PV thrombosis within 30 d post PD was observed in both end‐to‐end (2/97) and graft (1/25) patients, respectively. However, since direct end‐to‐end anastomosis is linked to better R0 resections,^18^ it is preferred even for up to 50‐mm resections by mobilizing the liver or performing a Cattell–Braasch maneuver.^19^ In this study, direct end‐to‐end anastomosis was done for 20 mm (Case 2), 40 mm (Cases 3 and 4), and 50 mm (Cases 7 and 8) resections, respectively. While grafts were used for 35 mm (Case 6), 40 mm (Cases 9, 10, and 11), 50 mm (Case 5), and 70 mm (Case 1) resections. R0 resections were achieved in all the nongraft and 83.3% (5/6) in the graft patients.

**FIGURE 3:**
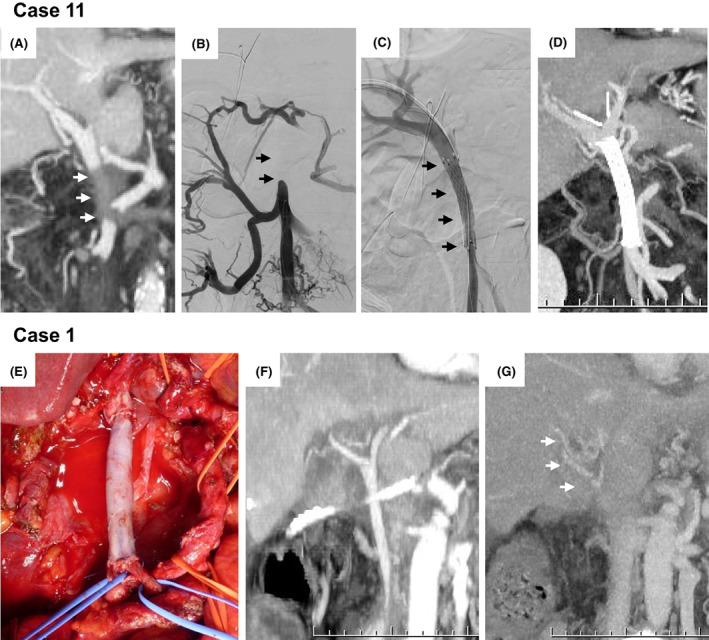
Case presentation (Cases 11 and 1). (A,B) PV thrombosis on POD1 (allows), (C) PV stent insertion on POD1 (allows), (D) patent PV stent on POD23, (E) inverted Y‐shaped reconstruction with venous graft, (F) PV graft patency on POD6, (G): PV stenosis with collateral veins on POD53 (allows). POD, postoperative day; PV, portal vein

The SMV/PV anastomosis remained patent in all five nongraft patients, while one graft patient (Case 11) had an immediate postoperative PV thrombosis and a percutaneous PV stent was placed on d 1 postsurgery (Figure 3A–D), as previously described.^20^ This acute thrombosis may have been caused by microthrombosis in distal veins due to a long interval between en‐bloc resection of the pancreatic head and SMV/PV segment to completing the inverted‐Y reconstruction, which might have led to prolonged small intestinal veins' congestion and induced the microthrombosis. It is also possible that the venous graft could have twisted on any of the two sites of SMV/PV anastomosis. Therefore, to prevent these problems the temporal intraoperative catheter‐bypass procedure of the PV proposed by Nakao^21^ may be useful when SMV/PV reconstruction requires a long time. It is also necessary to check the venous graft for torsion. Stent patency was confirmed on dynamic CT done 23 d later. In Case 1, SMV/PV anastomosis stenosis with significant collateral vein formation was observed on CT 2 mo post‐PD, as shown in Figure 3D–F, and we deduced that extensive dissection of the hepatoduodenal ligament, so that a longer SMV/PV segment could be resected (70 mm) and postoperative cholangitis could explain the cause of the stenosis, as previously reported.^22^, ^23^ These results are similar to other reports, Dua et al^18^ analyzed the outcomes in 90 post‐PD patients and performed SMV/PV reconstructions as follows: (i) longitudinal venorrhaphy in 17/90; (ii) transverse venorrhaphy in 9/90; (iii) primary end‐to‐end in 28/90; (iv) patch venoplasty in 17/90); and (v) interposition graft in 19/90. All patients in the end‐to‐end and transverse venorrhaphy groups remained patent, while thrombosis was seen in 16 (18%); longitudinal venorrhaphy (4/17), patch venoplasty (5/17), and interposition graft (7/19) patients, respectively.

However, our technique has some shortcomings that include risk for increased intraoperative bleeding, small intestinal veins' congestion, and microthrombosis in the distal veins due to prolonged venous clamping time, PV thrombosis or stenosis, and leg swelling. As a limitation, it may not be applied in PDAC patients where more than two distal branches of the SMV are involved. The main pitfalls include easy tearing of the fragile walls of the distal veins and increased operation time due to the surgical steps needed to complete the reconstruction.

Nonetheless, based on the overall perioperative outcomes of our 11 patients, which are comparable to previous reports,^11^, ^17^, ^18^ it is feasible to safely reconstruct the SMV/PV using the inverted Y‐shaped technique during PD for PDAC.

## 5 CONCLUSION

The inverted Y‐shaped technique can safely be used to reconstruct the SMV/PV in appropriately selected PD cases involving long‐segment SMV/PV axis encasement.

## AUTHOR CONTRIBUTIONS

Benson Kaluba: study concept and design, acquisition of data, and drafting of the article. Naohisa Kuriyama: study concept and design, study supervision, critical revision of the article. Takahiro Ito: study concept and design, acquisition of data. Akihiro Tanemura: study concept and design. Shugo Mizuno: study concept and design, study supervision.

## FUNDING INFORMATION

This research did not receive any specific grants from funding agencies in the public, commercial, or not‐for‐profit sectors.

## CONFLICT OF INTEREST

The authors declare no conflicts of interest for this article.

## ETHICAL APPROVAL

Approval of the research protocol: The study protocol was reviewed and approved by the Medical Ethics Committee of Mie University Hospital (No. 2857).

Informed Consent: This study was informed to all participants by opt‐out on the website of our institution instead of obtaining written consent forms from the participants because of the observational study.

Registry and the Registration No. of the study/trial: N/A.

Animal Studies: N/A.

## Supporting information


Video S1:

Video S2:

Video S3:

## Data Availability

Data supporting the findings of this study are available from Naohisa Kuriyama upon reasonable request.
